# Urinary Concentrations of Non-Nutritive Sweeteners and Breast Cancer: A Case-Cohort Analysis of the Moli-sani Study

**DOI:** 10.3390/nu18182958

**Published:** 2026-09-09

**Authors:** Marialaura Bonaccio, Augusto Di Castelnuovo, Simona Costanzo, Claudia F. Martinez, Emilia Ruggiero, Teresa Panzera, Simona Esposito, Amalia De Curtis, Sara Magnacca, Jacopo Troisi, Chiara Cerletti, Maria Benedetta Donati, Giovanni de Gaetano, Licia Iacoviello

**Affiliations:** 1 Research Unit of Epidemiology and Prevention, IRCCS NEUROMED, 86077 Pozzilli, IS, Italy; marialaura.bonaccio@moli-sani.org (M.B.); simona.costanzo@moli-sani.org (S.C.); claudianutre@gmail.com (C.F.M.); emilia.ruggiero@moli-sani.org (E.R.); teresa.panzera@moli-sani.org (T.P.); simona.esposito@moli-sani.org (S.E.); amalia.decurtis@moli-sani.org (A.D.C.); sara.magnacca@moli-sani.org (S.M.); chiara.cerletti@moli-sani.org (C.C.); mbdonati@moli-sani.org (M.B.D.); giovanni.degaetano@moli-sani.org (G.d.G.); licia.iacoviello@moli-sani.org (L.I.); 2 Research Center in Epidemiology and Preventive Medicine (EPIMED), Department of Medicine and Surgery, University of Insubria, 21100 Varese, VA, Italy; 3 Department of Medicine and Surgery, LUM University “Giuseppe Degennaro”, 70010 Casamassima, BA, Italy; 4 Theoreo Srl, 84090 Montecorvino Pugliano, SA, Italy; troisi@theoreosrl.com

**Keywords:** urinary non-nutritive sweeteners concentrations, sucralose, breast cancer, case-cohort study

## Abstract

**Background:** Non-nutritive sweeteners (NNS) are chemical substances developed by the food industry to reduce sugar and calories while preserving sweetness. Epidemiological studies have indicated a potential increased risk of certain cancers, including breast cancer (BC), associated with NNS consumption. However, the evidence has exclusively relied on self-reported dietary information rather than objective measurements of NNSs. We examined the association of urinary concentrations of NNS with BC risk in women from the large Moli-sani Study. **Methods:** Using a case-cohort design, a sample of 988 middle-aged women (mean age 55 ± 12 y) was randomly selected as a sub-cohort and compared to 272 women with incident BC (38 premenopausal and 234 postmenopausal). A total of 6 commonly used NNSs (i.e., aspartame, acesulfame, saccharin, sucralose, cyclamate, and steviol glycosides) were measured through liquid chromatography–mass spectrometry in spot urine samples (creatinine-adjusted concentrations; µg/mg) collected at baseline (2005–2010). The multivariable hazard ratios (HRs) were estimated using weighted Cox regression models for the case-cohort design, with each NNS modelled as a binary variable (i.e., presence vs. absence). The sum of these values produced a total urinary NNS score, which could range from 0 to 6, and was further categorised as low (0–1) or high (2–5). **Results:** The predominant NNS detected in urine was saccharin (E954), contributing to 77% of total NNSs, followed by steviol glycosides (E960;11%). In multivariable-adjusted Cox analyses, neither individual NNSs nor total NNS urinary concentrations were associated with overall BC risk (HR = 1.09; 95% CI 0.80–1.48 for high vs. low). However, the presence of sucralose (E955) was directly linked to premenopausal BC (HR = 5.90; 95% CI 1.44–24.20; *p* = 0.014), although this estimate was based on only 38 cases and was imprecise. **Conclusions:** Urinary NNS concentrations were not associated with overall BC risk. The observed association between urinary sucralose and premenopausal BC should be considered exploratory and hypothesis-generating and requires independent replication.

## 1. Introduction

Diets high in added sugars have been proposed as major contributors to the global epidemic of obesity and cardiometabolic disease, which has dramatically increased over recent decades [1,2,3]. Dietary factors and adiposity may also be relevant to breast cancer risk. Specific dietary components, including fatty acids, dietary fibre, and sugars, have been investigated in relation to breast cancer risk, while evidence from the Global Cancer Update Programme supports the role of overall healthy dietary and lifestyle patterns and the maintenance of a healthy weight in breast cancer prevention [4,5].

Non-nutritive sweeteners (NNS) are substances used to provide sweet taste with little or no caloric contribution and are commonly used to replace sugar in foods and beverages [6]. They can be consumed as tabletop products, alone or in combination, or used as ingredients in a wide range of foods and beverages, including soft drinks, dairy products, desserts, confectionery, cereals, and other processed foods.

Consequently, exposure to individual sweeteners may vary substantially according to dietary habits and product formulations. Their use has been proposed as a strategy to reduce sugar and energy intake and to support weight loss or weight maintenance. Randomised controlled trials have generally shown that replacing sugar with NNSs may reduce energy intake and support modest reductions in body weight, particularly when used as a substitute for sugar-containing foods and beverages [7]. However, evidence regarding their potential adverse metabolic effects remains heterogeneous and may depend on the specific sweetener, dose, comparator, and study population [8].

Aspartame (E951), for example, has been classified as possibly carcinogenic to humans by the International Agency for Research on Cancer [9,10]. Experimental studies have suggested potential effects of NNSs on oxidative stress, DNA damage, genotoxicity, cell proliferation and apoptosis, as well as on the gut microbiota and related inflammatory pathways [11,12,13,14], which are relevant to cancer onset [15,16]. However, findings across experimental models remain inconsistent, and their relevance to human cancer risk is uncertain.

A short-term randomised controlled trial in healthy adults reported sweetener-specific alterations in the gut microbiome and metabolome, while impaired glycemic responses were observed primarily following saccharin and sucralose supplementation [17]. However, other recent randomised studies have not observed adverse effects of NNSs on glycemic control or gut microbiota composition [18,19,20].

Observational cohort studies [7,21] have reported associations between NNS consumption and certain cancer outcomes, although results remain conflicting [22].

Importantly, these studies have largely relied on self-reported dietary information, which may introduce exposure misclassification and may not accurately capture exposure to individual NNSs from different dietary sources.

The primary aim of this study was to investigate the association between urinary concentrations of NNSs and incident breast cancer among women participating in the Moli-sani Study. As a secondary outcome, we examined the association between urinary concentrations of NNSs and selected biomarkers of glucose metabolism, inflammation, and renal function to explore potential biological pathways linking NNS exposure to chronic disease, including breast cancer.

## 2. Methods

### 2.1. Study Design and Population

The Moli-sani Study is a population-based cohort study established in 2005–2010 with an enrolment of 24,325 men and women (aged ≥35 y) randomly recruited from the general population of Molise, a southern Italian region. The primary aim was to investigate genetic and environmental risk factors in the onset and progression of cardiovascular and cerebrovascular disease, and cancer. Exclusion criteria were pregnancy at the time of recruitment, mental or decision-making impairments, current polytrauma or coma, or refusal to sign the informed consent. Further details of the study are documented elsewhere [23].

In the Moli-sani Study, a case-cohort design was utilised to efficiently evaluate urinary AS concentrations within a reference subcohort, which serves as the comparator group for various disease outcomes. Among cancer-free women with complete health and dietary data at baseline (*n* = 11,443), 272 new cases of BC were identified during a median follow-up of 13.2 years. Among the participants, 988 were selected by unstratified simple random sampling, independently of subsequent breast cancer status. The resulting subcohort sampling fraction was 988/11,443 = 0.0863 (8.63%), corresponding to an inverse probability weight of 11.58. The final sample consisted of 988 subcohort members and 272 incident BC cases (38 premenopausal BC; 234 postmenopausal BC), among whom 26 incident BC cases overlapped with the subcohort, as a design feature of a case-cohort study (Supplementary Figure S1).

This sample size provides 80% power (α = 0.05) to detect hazard ratios greater than 1.40 or lower than 0.70 per 1 standard deviation increase in continuous exposures.

### 2.2. Exposure Assessment

A total of 6 commonly used NNSs (i.e., aspartame, acesulfame, saccharin, sucralose, cyclamate, and steviol glycosides) were measured through liquid chromatography–mass spectrometry in participants’ 3 h first-morning urine spot samples (creatinine-adjusted concentrations; µg/mg) collected at the baseline visit (2005–2010) and stored at −80 °C at the Neuromed Biobanking Centre. Urinary sweetener concentrations were normalised to urinary creatinine and expressed as µg/mg creatinine to reduce variability attributable to urine dilution in spot samples. This normalisation was intended to account for differences in urinary concentration and should not be interpreted as correcting for compound-specific renal handling. The analytical method was validated using matrix-matched calibration curves and included assessment of linearity, limits of detection (LOD) and quantification (LOQ), recovery, and repeatability. Analyte-specific validation parameters and the mathematical procedures used to derive LOD and LOQ are reported in the Supplementary Methods and Supplementary Table S1.

Full details of sample preparation, chromatographic and mass-spectrometric conditions, analytical validation, quality control procedures, and handling of measurements below analytical thresholds are provided in the Supplementary Methods.

The analytical panel targeted the intact parent sweetener compounds. This choice was made to provide compound-specific measurements that could be assessed consistently across participants. Metabolites were not included because, for several sweeteners, downstream products are not exposure-specific or are strongly influenced by individual metabolic pathways. Accordingly, the parent compound measurements were used as comparative urinary exposure markers rather than as comprehensive measures of total metabolic exposure.

### 2.3. Outcome Assessment

The Moli-sani Study cohort was followed up for BC incidence from study entry through December 2020. Incident BC cases, including both fatal and non-fatal events, were identified through direct linkage with hospital discharge records using ICD-9-CM code 174.

Each case was validated by reviewing corresponding medical records that indicated BC as a diagnosis, with confirmation from histopathological reports. Fatal BC cases were further verified through linkage with the regional mortality registry (ReNCaM), based on death certificates listing ICD-9 code 174 as the primary cause of death.

Premenopausal BC was defined as cases diagnosed before the age of 50, reflecting the median age of menopause onset within this cohort. Cases diagnosed at age 50 or older were classified as postmenopausal BC, as previously done in this cohort [24].

### 2.4. Dietary Data Collection

Participants’ dietary intake over the preceding 12 months was assessed at baseline using a semi-quantitative food frequency questionnaire (FFQ) administered by trained interviewers. This FFQ was derived from the European Prospective Investigation into Cancer and Nutrition (EPIC) and had been previously validated and adapted for the Italian population [25,26]. A dedicated software program [25] linked the reported frequency and portion size of each food item to the Italian Food Tables [27], enabling the estimation of daily intake of energy, macronutrients, and micronutrients. Overall diet quality was evaluated using the Mediterranean Diet Score (MDS) developed by Trichopoulou et al. [28]. Foods were also classified according to the NOVA food-processing classification [29], and ultra-processed food consumption was defined as the intake of foods classified within NOVA group 4.

### 2.5. Biomarkers Assessment

Experimental evidence has identified several potential pathways through which NNSs may impact human health [11,12,13]. To explore this further, we investigated the association of urinary AS concentrations with selected biomarkers reflecting different underlying pathways common to chronic disease incidence and progression [30,31,32].

Blood samples were collected at baseline (2005–2010) from participants who had fasted overnight and abstained from smoking for at least 6 h. Blood glucose levels were measured in fresh serum samples using enzymatic reaction methods on an automatic analyser (ILab 350, Instrumentation Laboratory, Milan, Italy). Quality control for blood glucose was maintained using two commercial standards, SeraChem^®^ 1 (normal levels) and SeraChem^®^ 2 (high pathological levels), with coefficients of variation (CVs) of 4.7% and 4.1%, respectively. The analyses were performed using an instrument manufactured by Instrumentation Laboratory S.p.A. (Milan, Italy).

High-sensitivity C-reactive protein (CRP) was measured in fresh serum samples using a particle-enhanced immunoturbidimetric assay (ILab 350, Instrumentation Laboratory, Milan, Italy), with quality control maintained through an in-house serum pool and commercial laboratory standard. The CVs for CRP were 5.5% and 4.2%, respectively. Hematocytometry analysis was performed within 3 h of blood collection using a cell counter (Coulter HMX, Beckman Coulter, Milan, Italy). Quality control was conducted with three standards: Abnormal 1 (Abn1) for high levels, Abnormal 2 (Abn2) for low levels, and Normal (Coulter HMX, Beckman Coulter). The CV for white blood cell (WBC) counts was 6.2%, 3.3%, and 3.0% for Abn I, Abn II, and Normal, respectively. Renal function markers (Cystatin C, Creatinine), insulin, and C-peptide were later measured in thawed samples stored in liquid nitrogen vapours at the Neuromed Biobanking Center. These measurements were part of the collaborative BiomarCaRE research project (EUFP7, HEALTH-F2-2011-278913), whose primary objective is to assess the value of established and emerging biomarkers for CVD risk prediction using data from 23 cohorts across Europe [30]. The Homeostasis Model Assessment of Insulin Resistance (HOMA-IR) was used to estimate insulin resistance, a hallmark of impaired glucose, based on fasting insulin (mU/L) and glucose levels (mg/dL) [33]. A HOMA-IR value greater than 2.5 suggests significant insulin resistance [34], and lower values reflect better insulin sensitivity. Kidney function was assessed through the estimated glomerular filtration rate (eGFR). The eGFR was calculated using the 2021 Chronic Kidney Disease Epidemiology Collaboration creatinine–cystatin C (eGFRcr-cys) equation [35]; impaired kidney function was defined as eGFRcr-cys values <60 mL/min/1.73 m^2^.

### 2.6. Assessment of Covariates

Baseline information on socio-demographic characteristics, lifestyle factors, and medical history was collected through interviewer-administered questionnaires.

Educational level was categorised based on the highest qualification attained: lower secondary education (≤8 years of study), upper secondary education (9–13 years), and postsecondary education (>13 years). Housing tenure was classified into three categories: renting, ownership of a single dwelling, or ownership of more than one dwelling. Marital status was grouped as married/in a couple, separated/divorced, single, or widowed.

Participants were categorised as never, current, or former smokers, with the latter defined as individuals who had not smoked for at least the previous 12 months. Leisure-time physical activity (PA) was evaluated using a structured interviewer-administered questionnaire [36] and expressed as daily energy expenditure in metabolic equivalent task hours (MET-h/day), covering activities such as sports, walking, and gardening.

Height and weight were measured, and body mass index (BMI) was calculated as kg/m^2^ and grouped into three categories, normal (<25 kg/m^2^), overweight (≥25 <30 kg/m^2^), and obese (≥30 kg/m^2^). Personal history of cardiovascular diseases (i.e., angina, myocardial infarction, revascularisation procedures, peripheral artery disease, and cerebrovascular events) was self-reported and confirmed by medical records and therapy at study entry. Participants were considered to have diabetes, hypertension, or hyperlipidaemia at baseline if they were taking disease-specific drugs. Reproductive health information, including menopausal status, use of hormonal contraceptives or hormone replacement therapy, age at menarche, parity, and family history of breast cancer, was self-reported during the baseline assessment.

### 2.7. Statistical Analysis

For statistical handling of continuous urinary concentration data, measurements below the validated analytical thresholds were treated as left-censored observations. Values below the LOD were replaced by LOD/√2, whereas detectable measurements below the LOQ were replaced by LOQ/√2. These substituted values were used for statistical data handling and do not represent directly quantified analytical concentrations. Analytical detectability itself was defined from the original measurement relative to the analyte-specific LOD.

Due to the skewed distribution of NNS concentrations and the high prevalence of non-detectable levels in this population, the exposures were modelled as a binary variable (presence vs. absence) to improve comparability and reduce misclassification. Therefore, binary variables were created for each NNS detected in urine (i.e., acesulfame, saccharin, sucralose, cyclamate, steviol glycoside, and aspartame), and we used 0 or 1 to indicate absence or presence according to a specified threshold. For saccharin, which followed a normal distribution, the median value of 0.29 µg/mg creatinine-adjusted was used as the cut-off. For all other NNSs, more than 50% of participants in the subcohort had concentrations below or close to the analyte-specific detection threshold. Therefore, exposure was modelled as detectable versus non-detectable, with values above the analyte-specific LOD classified as detectable and values at or below the LOD classified as non-detectable. The cut-offs used were as follows: acesulfame, 0.0071 µg/mg creatinine-adjusted; sucralose, 0.0084 µg/mg creatinine-adjusted; cyclamate, 0.011 µg/mg creatinine-adjusted; steviol glycoside, 0.0092 µg/mg creatinine-adjusted; and aspartame, 0.0043 µg/creatinine-adjusted creatinine. These thresholds were derived based on concentrations observed in the sub-cohort.

To evaluate overall urinary NNS exposure, we summed the binary values for each of the six NNSs (0 = absence/non-detectable; 1 = presence/detectable) to obtain a score potentially ranging from 0 to 6, with higher values indicating the detection of a greater number of NNSs in urine. This score was then categorised a priori into low (0–1 NNSs detected) and high (2–5 NNSs detected) exposure groups. This categorisation was based on the distribution of the score in the subcohort and was intended to distinguish participants with detection of one or fewer NNS from those with detection of multiple NNSs.

The baseline characteristics of the subcohort, stratified by level of total urinary concentrations of NNSs, were described as mean ± standard deviation (SD) for continuous variables, and frequencies and percentages for categorical variables. Differences in the distribution of baseline covariates by level of total urinary concentrations of NNSs were calculated using generalised linear models adjusted for age and energy intake (GENMOD procedure for categorical variables and GLM procedure for continuous variables in SAS 9.4 software). Associations between urinary NNS concentrations (high vs. low) and the incidence of overall breast cancer and its subtypes were estimated using weighted Cox proportional hazards regression models adapted to the case-cohort design. Hazard ratios (HRs) and 95% confidence intervals (CIs) were obtained using Barlow inverse probability weights, based on the subcohort sampling fraction, with robust sandwich variance estimation to account for the case-cohort sampling and for the multiple observation intervals contributed by subcohort cases. Subcohort members contributed to the risk sets throughout follow-up with a weight equal to the inverse of the sampling fraction, whereas cases outside the subcohort entered the risk set only at their failure time with unit weight. The 26 cases who were also members of the subcohort were not counted as additional participants. Their follow-up was split according to the Barlow method: they received the inverse sampling fraction weight before breast cancer diagnosis and unit weight at the event time. Participants were followed from study entry until breast cancer diagnosis, death, emigration, loss to follow-up, or the end of follow-up, whichever occurred first. Death before breast cancer diagnosis was treated as a competing event and censored at the date of death; therefore, the weighted Cox models estimated cause-specific hazard ratios. Potential confounders (measured at baseline) were defined a priori and identified based on the existing literature, rather than relying on statistical criteria [37]. The proportional hazards assumption was assessed by including interaction terms between each NNS exposure and log-transformed follow-up time in the weighted Cox models, with no indication of departure from proportionality.

Two models were ultimately fitted: (a) Model 1 included age (continuous) and energy intake (Kcal/d); (b) Model 2 included the same information as Model 1 and further controlled for educational level (up to lower secondary, upper secondary, post-secondary); housing tenure (rented, ownership of one dwelling, and ownership of more than one dwelling); place of residence (urban/rural); marital status (married/in couple; unmarried); occupational social class (high: professional/managerial and skilled non-manual workers; medium: skilled manual workers; low: semi-skilled/unskilled and unemployed/unclassified); smoking status (never, current, former smokers); BMI (normal weight, <25 kg/m^2^; overweight, 25–29.9 kg/m^2^; obese, ≥30 kg/m^2^), leisure-time physical activity (continuous); number of chronic diseases (≤1 or >1); menopausal status (no/yes); use of oral contraceptives (no/yes); use of hormone replacement therapy (no/yes); number of children (i.e., 0; 1–2: and >2); age at menarche (≥11 or <11 years of age); family history of BC (no/yes); and the Mediterranean Diet Score.

Each individual NNS exposure was entered separately into Models 1 and 2. As a sensitivity analysis, we additionally adjusted Model 2 for ultra-processed food consumption expressed as the percentage of total energy intake to assess whether the observed associations were influenced by potential confounding by UPF consumption. Predefined subgroup analyses were conducted according to BMI, age, and family history of breast cancer. Analyses by BC subtype (i.e., pre- and postmenopausal BC) were also conducted to determine whether associations varied by menopausal status. Associations of individual urinary NNS concentrations (high vs. low) with selected biomarkers (modelled as continuous variables) were assessed through multivariable-adjusted regression models. To maximise data availability, missing data were handled using a regression-based imputation method (single imputation) with the PROC MI procedure in SAS (see Supplementary Figure S1). The data analysis was generated using SAS/STAT software, version 9.4 (SAS Institute Inc., Cary, NC, USA).

## 3. Results

**Figure 1:**
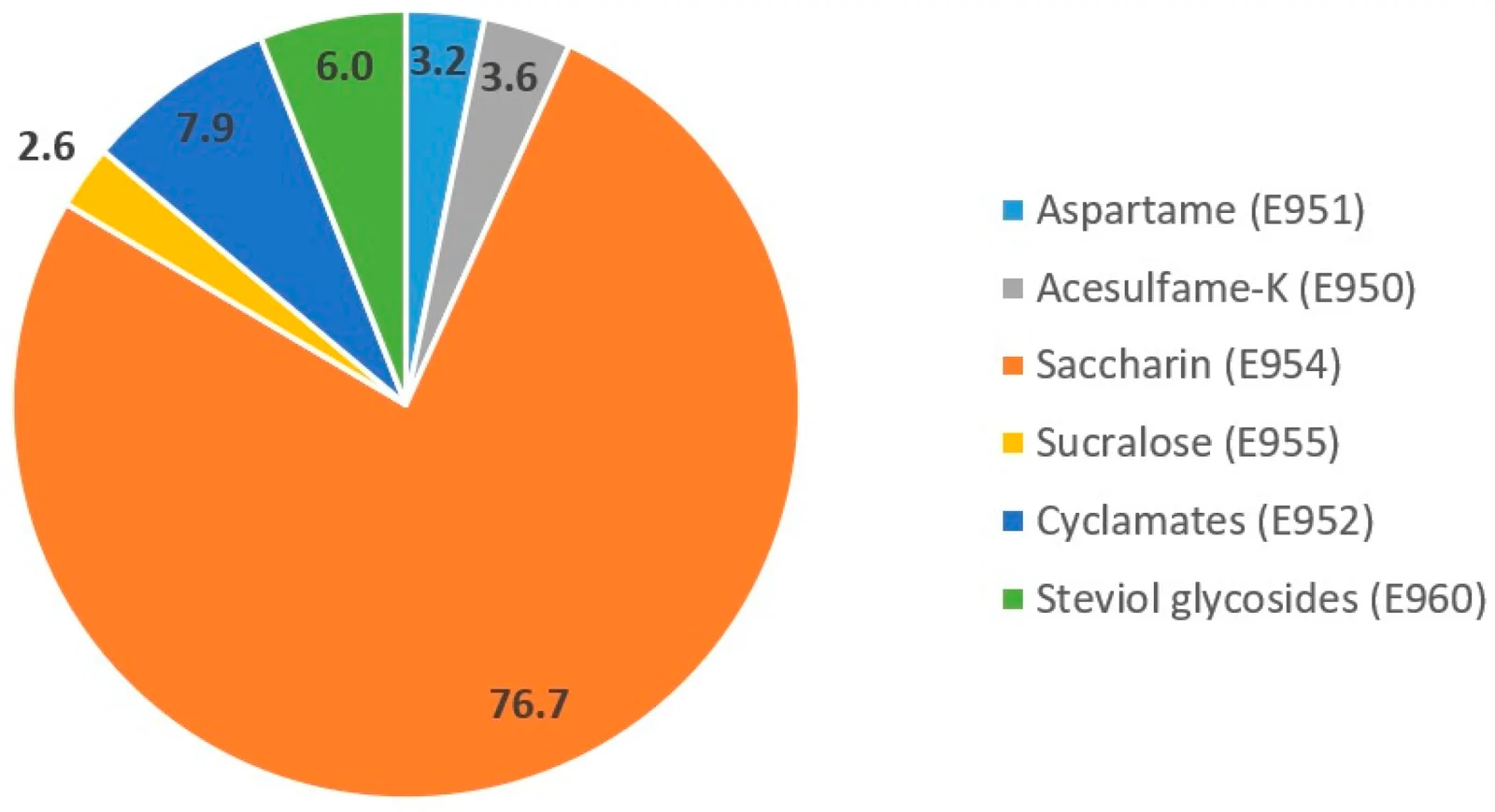
Relative contribution of each individual non-nutritive sweetener to the total urinary concentrations of non-nutritive sweeteners (percentage), Moli-sani Study, Italy 2005–2010.

In subcohort participants (mean age 54.5, SD 11.6), saccharin was the predominant NNS detected in urine, accounting for 76.7% of the total urinary NNS concentration, followed by cyclamate (E952; 7.9%) and steviol glycosides (E960; 6.0%) (Figure 1). Descriptive statistics for urinary concentrations of individual NNSs, their relative contribution to total urinary NNS concentrations, and their main dietary sources in the Italian market are reported in Supplementary Table S2. Participants with higher urinary NNS (i.e., high total NNS score) exhibited lower socioeconomic status, reflected by indicators of educational level, housing tenure, and occupational classes, and were less likely to reside in urban areas compared to those with lower urinary NNS concentrations. Participants with higher urinary concentrations of NNSs were more likely to be smokers and had a higher BMI (Table 1). The dietary characteristics of study participants are displayed in Table 2. Higher urinary concentrations of NNSs were inversely associated with energy intake and directly with consumption of artificial sweeteners, while no associations were observed with dietary scores, food groups or macronutrients (Table 2).

**Figure 2:**
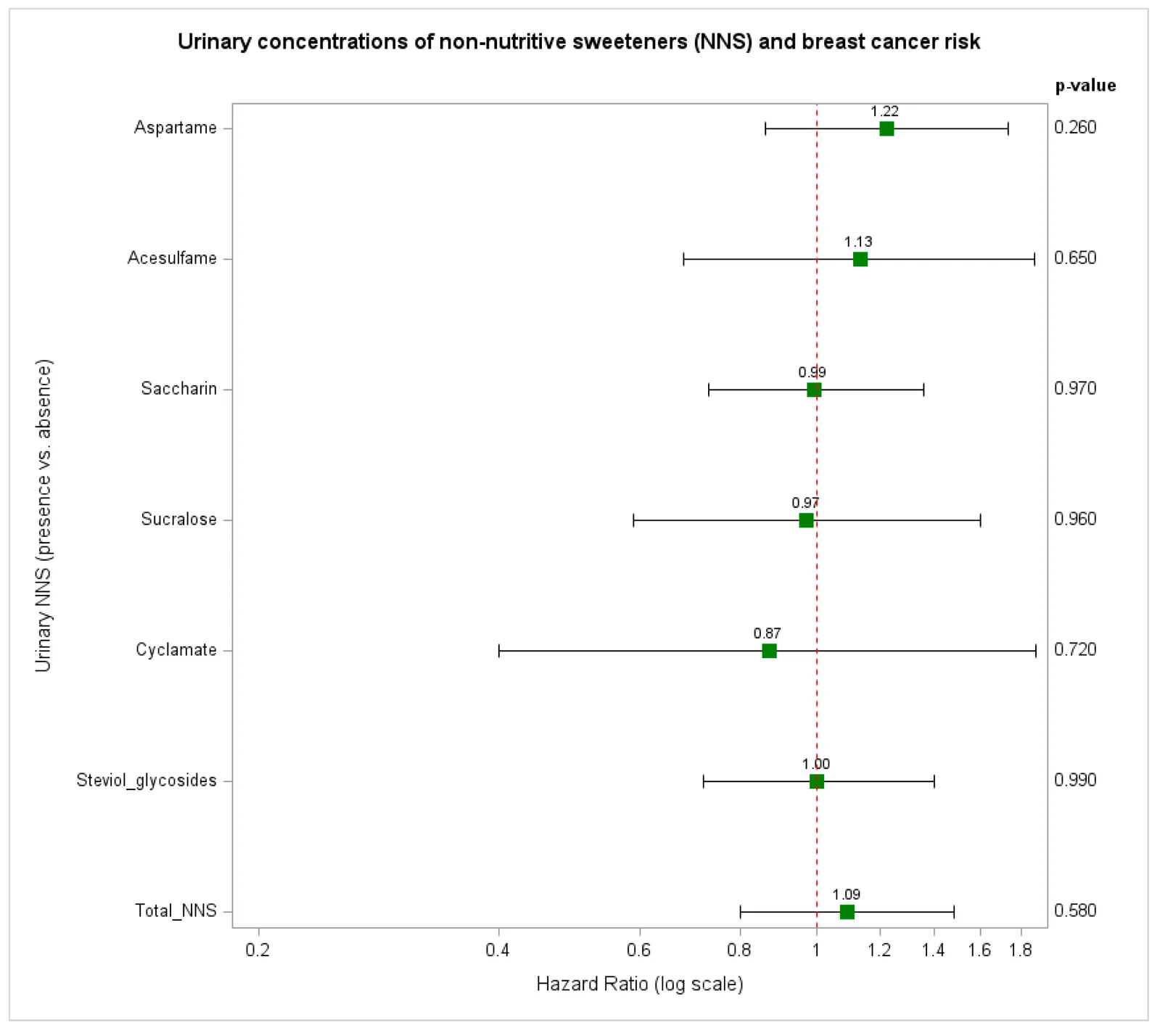
Association of urinary concentrations of non-nutritive sweeteners (NNS) and breast cancer risk, Moli-sani Study, Italy 2005–2010. Hazard ratios (HRs) and 95% confidence intervals (CIs) were estimated using weighted Cox proportional hazards regression models adapted to the case-cohort design. Each individual NNS was entered separately into the model. Models were adjusted for age, energy intake, educational level, housing tenure, place of residence, marital status, occupational social class, smoking status, body mass index, leisure-time physical activity, number of chronic diseases, menopausal status, oral contraceptive use, hormone replacement therapy, number of children, age at menarche, family history of breast cancer, and the Mediterranean Diet Score. For each NNS, exposure was modelled as presence versus absence according to the analyte-specific cut-off. The total urinary NNS score was modelled as high (2–5) versus low (0–1). Red dashed line indicates HR = 1.0 (null value).

In multivariable-adjusted Cox regression models, neither total urinary concentrations (high vs. low) nor individual NNSs were associated with risk of breast cancer (Table 3, Model 2, and Figure 2).

**Figure 3:**
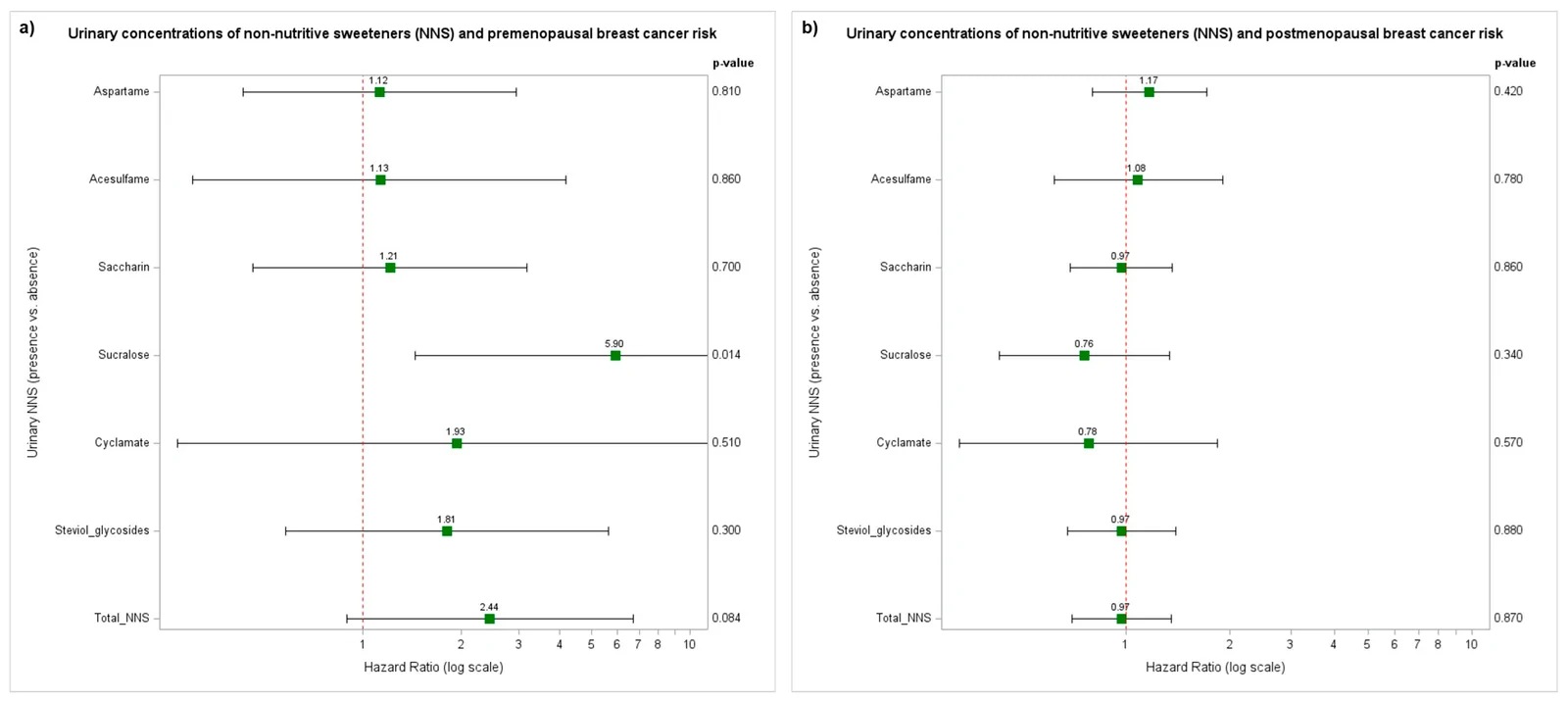
Association of urinary concentrations of non-nutritive sweeteners (NNS) and (**a**) premenopausal and (**b**) postmenopausal breast cancer risk, Moli-sani Study, Italy 2005–2010. Hazard ratios (HRs) and 95% confidence intervals (CIs) were estimated using weighted Cox proportional hazards regression models adapted to the case-cohort design. Each individual NNS was entered separately into the model. Models were adjusted for age, energy intake, educational level, housing tenure, place of residence, marital status, occupational social class, smoking status, body mass index, leisure-time physical activity, number of chronic diseases, oral contraceptive use, hormone replacement therapy, number of children, age at menarche, family history of breast cancer, and the Mediterranean Diet Score. For each NNS, exposure was modelled as presence versus absence according to the analyte-specific cut-off. The total urinary NNS score was modelled as high (2–5) versus low (0–1). Red dashed line indicates HR = 1.0 (null value).

Urinary concentrations of sucralose were associated with an increased risk of premenopausal BC (HR = 5.90; 95% CI 1.44–24.20; *p*-value = 0.014) (Figure 3a).

Data are expressed as hazard ratios (HRs) with 95% confidence intervals (95% CIs), obtained from multivariable-adjusted weighted Cox regression models accounting for the case-cohort design. Model 1 was adjusted for age and energy intake. Model 2 was additionally adjusted for educational level, housing tenure, marital status, occupational class, place of residence, smoking status, leisure-time physical activity, body mass index, number of chronic diseases, menopausal status, hormone replacement therapy, oral contraceptive use, number of children, family history of breast cancer, and the Mediterranean Diet Score. Menopausal status was not included in the analyses stratified by premenopausal and postmenopausal breast cancer incidence. However, after controlling for multiple comparisons using the false discovery rate (FDR) approach (Supplementary Table S3), the strength of the evidence was attenuated (FDR-adjusted *p* = 0.084), suggesting that this finding should be interpreted with caution and considered exploratory. No associations between individual or total urinary excretion of NNSs and postmenopausal BC were observed (Table 3, Model 2, and Figure 3b). Urinary excretions of sucralose were not associated with any of the markers here analysed (Table 4). Additional adjustment for ultra-processed food consumption did not materially change the associations between urinary NNS and either overall or subtype-specific breast cancer risk (Supplementary Table S4).

In subgroup analyses, no consistent differences in the associations between urinary NNS and breast cancer risk were observed according to body mass index, age, or family history of breast cancer (Supplementary Table S5). Although some subgroup-specific estimates differed in magnitude, confidence intervals were wide and largely overlapped, providing no evidence of a consistent pattern of effect modification.

## 4. Discussion

This study examined the association between urinary biomarkers of NNS and the risk of BC in a large cohort of middle-aged women from the Moli-sani Study. Our findings suggest that, overall, there was no association of total or individual urinary NNS concentrations with the risk of developing BC. However, an association was observed between urinary concentrations of sucralose and increased risk of premenopausal BC, although it should be considered exploratory in nature. It is also possible that the lack of association observed for total NNS and other individual compounds reflects, at least in part, the limited statistical power of our study to detect modest effect sizes; thus, small associations cannot be excluded given the available sample size.

The epidemiological evidence investigating the relationship between NNS and BC risk remains limited and inconsistent. A meta-analysis including over 300,000 participants from two case-control studies and three cohort studies indicated no association between dietary sweetener consumption and BC incidence [38]. However, substantial heterogeneity was observed among the included studies, particularly regarding the types of exposure (e.g., sweeteners from beverages and/or consumption of sweetener packets) and the follow-up length.

Epidemiological studies examining the relationship between NNS and overall cancer risk also yielded mixed results. In a meta-analysis summarising findings from cohort studies published up to 2022 [39], only 4 out of the 14 records evaluated NNS consumption by combining self-reported intake of NNS-containing beverages with AS packet use [40,41,42]. In contrast, the study by Debras and colleagues [6] uniquely relied on quantitative AS intakes from all dietary sources, distinguishing the different types of sweeteners.

While the overall findings of this meta-analysis indicated a lack of association between NNS intake and cancer incidence (HR = 1.04; 95% CI 0.99–1.08; heterogeneity = 34.5%), when pooling results from these four studies, an increased risk of cancer incidence was observed (HR= 1.09; 95% CI 1.01–1.18; heterogeneity = 0.0%) [39]. It is relevant to note that analysing exposure to NNSs primarily from NNS-containing beverages provides limited information, since NNSs are found in a variety of processed foods and beverages, including flavoured yoghurts, baked goods, cereals, sugar-free gum, protein powders, and condiments.

Although evidence from observational studies generally suggest that typical NNS intake levels may not represent a significant carcinogenic risk, findings from animal models and biochemical studies indicated potential pathways through which NNSs could impact cancer risk, including DNA damage and genotoxicity [14], dysbiosis and glucose intolerance [11], endocrine disruption [43], and inflammation [44], which have also been observed in humans [45].

**Figure 4:**
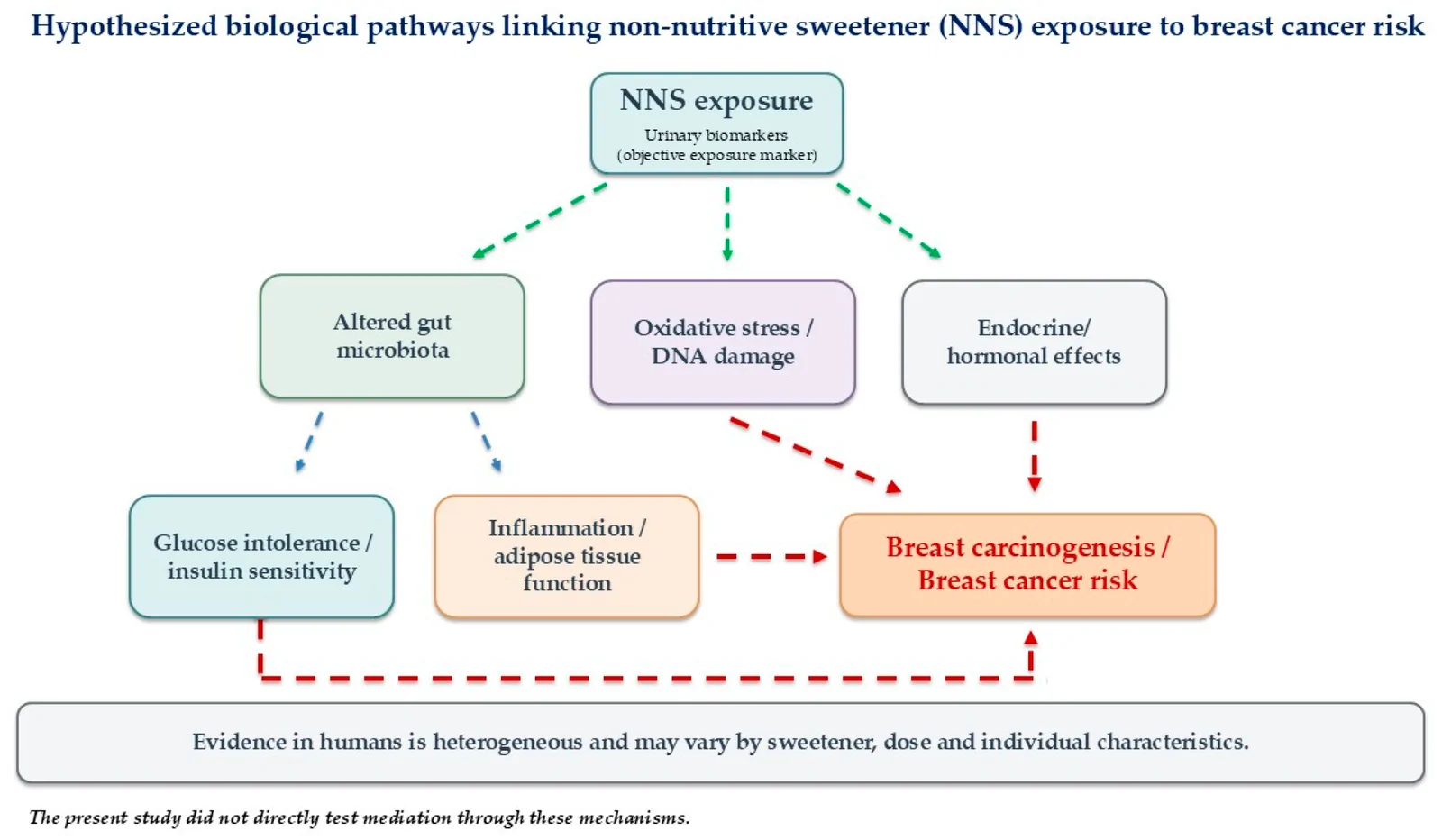
Selected hypothesised biological pathways potentially linking non-nutritive sweetener (NNS) exposure to breast cancer risk.

NNSs may potentially influence cancer-related pathways through metabolic and tissue-specific mechanisms, including insulin sensitivity, adipose tissue function, inflammation, and gut microbiota [17,45,46]. However, evidence in humans remains heterogeneous and appears to vary according to the specific sweetener, dose, and individual characteristics [8,46]. These pathways are potentially relevant to breast carcinogenesis, given the established role of adiposity, insulin resistance, and chronic inflammation (Figure 4).

In our study, however, adjustment for BMI did not materially alter the associations, and no consistent differences were observed across BMI categories.

The exploratory association observed between urinary sucralose and premenopausal BC warrants further research, given that sucralose is one of the most widely used NNSs globally. Sucralose is 600 times sweeter than sucrose and is used in over 4500 products [47]. It has long been considered a safe substance, and the U.S. Food and Drug Administration has affirmed its safety for consumption by both children and individuals with diabetes [48]. A systematic review of long-term carcinogenicity studies in animal models concluded that there is no evidence of carcinogenic potential for sucralose [49]. However, recent experimental studies examining the impact of sucralose on cancer-related pathways have yielded mixed results, with some studies suggesting potential biological effects that could influence carcinogenesis.

Studies in murine models indicated that sucralose increases the risk of malignant tumours and hematopoietic neoplasia [50], while also negatively affected the immune system through T-cell function disruption [51]. Additionally, a sucralose metabolite, sucralose-6-acetate, has been found to induce the expression of genes linked to inflammation, oxidative stress, and cancer [14].

Although short-term studies have not found significant effects of sucralose consumption on gut health [52], long-term animal research has linked sucralose to disturbances in the gut microbiome and increased inflammation [53]. More recently, translational evidence has suggested that sucralose may impair antitumour immunity through microbiome-mediated mechanisms, further supporting the hypothesis that it may influence tumour-related biological pathways, although in the context of cancer progression rather than initiation [54].

A randomised clinical trial involving healthy volunteers who received different NNSs (i.e., saccharin, sucralose, aspartame, and stevia) for 2 weeks at doses below the acceptable daily intake reported impaired glycemic responses following saccharin and sucralose supplementation [17], although these findings have not been consistently replicated, nor on glycemic control or gut microbiota composition [18,19,20]. Another human trial found that the intake of sucralose alongside another carbohydrate (i.e., maltodextrin) disrupts the gut–brain regulation of glucose metabolism [55].

However, these findings should be interpreted cautiously, as a subsequent reanalysis raised concerns about the choice of comparator and suggested that the observed effects could be attributable to the carbohydrate component rather than sucralose itself [56].

In our study, the observed association with premenopausal BC incidence, but not postmenopausal BC, suggests that hormonal differences may influence the relationship between NNS exposure, specifically sucralose, and cancer risk. Our data are consistent with findings from the Nutrinet Santè cohort [6], which indicate a trend of increased premenopausal BC risk, but not of postmenopausal BC risk, among participants reporting sucralose consumption compared to non-consumers (HR = 1.26; 95% CI 0.97–1.64; *p* value = 0.089). Interestingly, saccharin was the predominant NNS detected in our urinary samples. Although urinary biomarkers and dietary intake estimates are not directly comparable, differences in the absorption, metabolism, and excretion of individual NNSs may substantially influence urinary profiles and contribute to discrepancies between dietary exposure patterns and urinary biomarker distributions [57,58]. Notably, recent studies in European populations have also identified saccharin, acesulfame-K, and cyclamate among the most frequently detected NNS [57,59].

Whether NNS exposure may influence treatment response, disease progression, or breast cancer recurrence remains largely unexplored and could not be addressed in the present study, which focused on incident BC among women who were cancer-free at baseline.

We did not find any association between urinary concentrations of sucralose and the biomarkers examined. As a result, we were unable to test whether the increased risk of premenopausal BC associated with urinary concentrations of sucralose could be explained through any of these pathways.

### Strengths and Limitations

A notable strength of this study is the use of liquid chromatography–mass spectrometry for an objective quantification of NNS metabolites in urine, thereby enhancing the accuracy of our exposure assessment independent of participants’ reporting biases. Indeed, previous research in this field relied on self-reported dietary data to determine NNS intake, which is susceptible to several limitations, including recall bias and inaccuracies in reporting, and individual variation in dietary habits. These factors can lead to misclassification of exposure and potentially skew the results.

Through the use of urinary biomarkers of NNS exposure, our study provides an objective measure that is independent of self-reported dietary information. However, urinary concentrations should not be interpreted as direct quantitative estimates of dietary intake, because they also reflect compound-specific absorption, metabolism, systemic distribution, and renal handling. Creatinine normalisation reduces variability attributable to urine dilution in spot samples but does not correct for differences in glomerular filtration, tubular secretion, or tubular reabsorption among individual sweeteners. Consequently, urinary concentrations may reflect both external exposure and compound-specific pharmacokinetic and renal processes. This limitation is further compounded by the use of a single baseline spot urine sample, which may not fully capture habitual long-term exposure.

However, we acknowledge the use of spot urine samples as a limitation, since it may not fully capture habitual exposure to NNS compared to 24 h urine collections. In addition, stool samples were not collected in this cohort, and therefore, intestinal exposure to poorly absorbed NNS and potential interactions with the gut microbiota could not be assessed. An additional consideration concerns the compound-specific metabolism of NNSs. The present analytical approach quantified the intact parent compounds and did not include downstream metabolites. This limits the ability to reconstruct total metabolic exposure but also reflects an intentional design choice aimed at maximising analytical specificity and comparability across participants. For some compounds, downstream metabolites are not specific to the sweetener itself. Aspartame is hydrolysed to aspartic acid, phenylalanine and methanol, all of which have multiple endogenous or dietary sources, making attribution of their urinary concentrations specifically to aspartame exposure problematic. Likewise, conversion of cyclamate to cyclohexylamine depends on intestinal microbial metabolism and may vary considerably across individuals. Metabolite concentrations could therefore reflect both exposure and interindividual metabolic phenotype.

The parent compound measurements used in this study should therefore be interpreted as standardised, compound-specific urinary exposure markers for comparative epidemiological analyses rather than as complete measures of total internal exposure or biologically active dose. The extent to which urinary parent concentrations reflect overall exposure is expected to differ among individual sweeteners.

Other strengths include the case-cohort design, which is particularly efficient in this context, as it enables the examination of a large cohort with incident BC cases while minimising the cost and time associated with a full cohort analysis. Furthermore, our analyses were adjusted for a wide range of confounders, boosting the validity of our findings.

Nevertheless, several limitations must be acknowledged. Firstly, although the urinary biomarkers offer an objective measure of NNS exposure, we were only able to assess exposure at a single time point (baseline). Repeated measurements of urinary NNS concentrations would provide a more comprehensive understanding of their role in BC risk. In addition, because most urinary NNS concentrations were highly skewed, with a large proportion of values at or below the limit of detection, the exposures were primarily modelled as binary variables. This approach may have resulted in some loss of information and precluded the assessment of potential dose–response or non-linear associations. However, similar categorisation approaches have been used in previous studies in this field [60,61]. Additionally, the sample size for BC incidence by menopausal status was relatively small, limiting the statistical power and precluding informative sensitivity analyses excluding early BC cases. Therefore, reverse causation cannot be excluded, as dietary habits, including NNS consumption, may have changed during a preclinical phase of BC. Moreover, although tumour hormone receptor data were available, the limited number of receptor-negative and HER2-positive tumours precluded adequately powered comparisons between receptor-defined subgroups. Another weakness is that menopausal status was defined using age (<50 years) as a proxy rather than direct information on menopausal status, consistent with previous analyses of the Moli-sani Study [19]. Another limitation is that the overall intake of NNSs in our population was relatively low, and this may contribute to the lack of a clear association with BC risk, as higher levels of exposure may be necessary to observe any potential effects. Previous studies that demonstrate links between NNSs and cancer risk may have involved populations with higher levels of NNS consumption, highlighting the need for future research in larger and more diverse samples with higher intake levels of NNSs for a comprehensive assessment of dose–response relationships. While our study provides valuable insights with its objective measurement of NNS exposure, it also underscores the necessity of considering intake levels when interpreting epidemiological results on NNSs and cancer risk. While we used a comprehensive set of markers that reflect various biological pathways associated with the onset of chronic diseases, we acknowledge that these markers do not encompass all possible pathways that might link sucralose to BC onset. Another limitation is that we did not apply a systematic adjustment for multiple comparisons, mainly due to the relatively small sample size. As a result, our findings, particularly those related to specific subgroups such as premenopausal women, should be interpreted as exploratory and hypothesis-generating rather than conclusive. Also, as this is an observational study, we cannot rule out the possibility of residual confounding. Although we adjusted for a large panel of potential confounders, including sociodemographic, lifestyle, and dietary factors, unmeasured variables may still have influenced the observed associations. Finally, caution is warranted in generalising these findings to other populations.

## 5. Conclusions

In conclusion, this study found no clear association between urinary NNS concentrations and overall BC incidence. However, the exploratory association observed between urinary sucralose concentrations and increased risk of premenopausal BC warrants further investigation. These findings do not support a clear association between NNS exposure and overall breast cancer risk, but highlight the need for independent replication in larger prospective studies with repeated biomarker measurements, quantitative dose–response assessment, and the evaluation of potential metabolic and gut microbiota pathways.

## Figures and Tables

**Table 1:** Baseline characteristics of the subcohort (*n* = 988) and total urinary concentrations of non-nutritive sweeteners (NNS).

		Total NNS in Urine (Score)		
	Subcohort	Low (0–1)	High (2–5)	*p*-Value
*N* of participants (%)	988 (100.0)	539 (54.6)	449 (45.4)	–
Age (years; means, SD)	54.5 (11.6)	55.0 (11.5)	53.9 (11.7)	0.12
Place of residence (urban)	690 (69.8)	74.8	63.9	<0.001
Educational level				0.047
Up to lower secondary	515 (52.1)	49.7	55.0	
Upper secondary	341 (34.5)	35.8	33.0	
Postsecondary	132 (13.4)	14.5	12.0	
Housing				0.012
Renting	89 (9.0)	7.2	11.1	
1 dwelling ownership	813 (82.3)	82.6	82.0	
>1 dwelling ownership	86 (8.7)	10.2	6.9	
Marital status (%)				0.43
Married	811 (82.1)	82.9	81.1	
Unmarried	177 (17.9)	17.1	18.9	
Occupational social class				0.0053
High	570 (57.7)	60.7	54.1	
Medium	134 (13.6)	14.5	12.5	
Low	284 (28.7)	24.8	33.4	
Smoking status				0.017
Non-smoker	640 (64.8)	68.3	60.6	
Current	203 (20.6)	18.7	22.7	
Former	145 (14.6)	13.0	16.7	
Body mass index (BMI)				0.11
Normal weight	340 (34.4)	36.7	31.6	
Overweight	350 (35.4)	34.1	37.0	
Obese	298 (30.2)	29.2	31.4	
BMI (kg/m^2^; means, SD)	27.8 (5.3)	27.4 (5.0)	28.2 (5.6)	0.020
Abdominal obesity	859 (86.9)	87.4	86.4	0.75
Diabetes	30 (3.0)	3.7	2.3	0.12
Hypertension	253 (25.6)	25.8	25.4	0.99
Hypercholesterolemia	72 (7.3)	7.1	7.6	0.75
History of CVD	39 (4.0)	3.3	4.7	0.22
Number of chronic diseases				0.91
0–1	915 (96.6)	92.8	92.4	
≥2	73 (7.4)	7.2	7.6	
Menopausal status	534 (54.1)	55.5	52.3	0.66
Oral contraceptive use (current or in the past)	285 (28.9)	28.9	28.7	0.60
Hormone replacement therapy (current or in the past)	56 (5.7)	5.8	5.6	0.99
Number of children				0.96
0	91 (9.2)	9.3	9.1	
1–2	587 (59.4)	58.6	60.4	
>2	310 (31.4)	32.1	30.5	
Age at menarche (years)				0.18
≥11	879 (89.0)	90.2	87.5	
<11	73 (7.4)	6.5	8.5	
Unascertained	36 (3.6)	3.3	4.0	
Family history of breast cancer	47 (4.8)	3.9	5.8	0.19

Data are reported as numbers and percentages, unless stated otherwise; means and *p*-values were obtained from general linear models adjusted for age and energy intake.

**Table 2:** Dietary characteristics (means, SD) of the subcohort (*n* = 988) and levels of urinary concentrations of non-nutritive sweeteners (NNS).

		Total NNS in Urine (Score)		
	Subcohort	Low (0–1)	High (2–5)	*p*-Value
*N* of participants (%)	988 (100.0)	539 (54.6)	449 (45.4)	–
Energy intake (kcal/d)	1935 (490)	1968 (505)	1896 (468)	0.016
Sweeteners intake (g/d)	0.06 (0.44)	0.03 (0.32)	0.10 (0.54)	0.012
Mediterranean Diet Score	4.34 (1.62)	4.37 (1.58)	4.30 (1.67)	0.52
Unprocessed/minimally processed food (weight ratio)	66.2 (9.7)	66.5 (9.5)	65.8 (9.9)	0.20
Processed culinary ingredients (weight ratio)	2.4 (0.9)	2.3 (0.8)	2.4 (0.9)	0.21
Processed food (weight ratio)	20.0 (7.8)	20.0 (7.6)	20.0 (8.0)	0.98
Ultra-processed food (weight ratio)	11.5 (6.7)	11.2 (6.5)	11.9 (6.9)	0.090
Fruit and nuts (g/d)	363 (203)	362 (201)	364 (204)	0.87
Vegetables (g/d)	160 (69)	163 (73)	156 (65)	0.073
Cereals (g/d)	183 (75)	184 (76)	183 (73)	0.78
Legumes (g/d)	29 (20)	30 (21)	28 (19)	0.086
Fish and seafood (g/d)	46 (26)	45 (24)	47 (27)	0.48
Milk and dairy products (g/d)	209 (123)	210 (126)	207 (120)	0.66
Meat and meat products (g/d)	94 (38)	92 (38)	96 (39)	0.053
Alcohol (g/d)	6.4 (10.1)	6.6 (10.3)	6.3 (10.0)	0.71
MUFA to SFA ratio	1.36 (0.28)	1.37 (0.29)	1.35 (0.28)	0.39
Carbohydrate, % TEI	49.3 (6.7)	49.3 (6.6)	49.2 (6.9)	0.91
Added sugar, g/d	9.9 (9.6)	10.0 (9.6)	9.9 (9.6)	0.95
Total sugar, g/d	92.5 (34.3)	92.3 (35.4)	92.7 (33.0)	0.79
Starch, g/d	146.2 (52.7)	146.4 (53.9)	146.0 (51.0)	0.87
Dietary Fibre, g/d	20.2 (6.3)	20.4 (6.3)	19.9 (6.1)	0.12
Protein, % TEI	16.8 (2.1)	16.8 (2.0)	16.8 (2.2)	0.50
Fat, % TEI	34.6 (5.2)	34.6 (5.2)	34.6 (5.2)	0.96
SFA, % TEI	12.5 (2.5)	12.4 (2.6)	12.5 (2.5)	0.70
SFA, g/d	27.0 (9.3)	27.0 (9.8)	27.0 (8.7)	0.93
MUFA, % TEI	16.6 (2.9)	16.6 (2.9)	16.5 (3.0)	0.73
Total PUFA, % TEI	3.6 (0.6)	3.6 (0.6)	3.6 (0.6)	0.54
Linoleic acid, g/d	6.1 (1.8)	6.1 (1.8)	6.1 (1.7)	0.66
α-Linolenic acid, g/d	1.04 (0.28)	1.04 (0.29)	1.04 (0.28)	0.92
Dietary cholesterol, mg/d	312 (101)	312 (103)	313 (99)	0.75
Sodium, mg/d	2159 (754)	2159 (756)	2159 (750)	0.99

MUFA = monounsaturated fats; PUFA = polyunsaturated fats; SD = standard deviation; SFA = saturated fats; TEI = total energy intake. Means and *p*-values were obtained from linear regression models adjusted for age and energy intake.

**Table 3:** Association between urinary concentrations of non-nutritive sweeteners (NNS) and breast cancer risk, overall and by menopausal status.

Urinary NNS	Subcohort, *n* (%)	BC Cases, *n* (%)	Model 1 HR (95% CI)	*p*-Value	Model 2 HR (95% CI)	*p*-Value
**Overall breast cancer (*n* = 272)**						
Aspartame (>0.004 µg/mg)	228 (23.1)	72 (26.5)	1.22 (0.89–1.68)	0.24	1.22 (0.86–1.73)	0.26
Acesulfame (>0.01 µg/mg)	97 (9.8)	28 (10.3)	1.15 (0.71–1.89)	0.57	1.13 (0.68–1.87)	0.65
Saccharin (>0.29 µg/mg)	495 (50.1)	145 (53.3)	1.05 (0.78–1.40)	0.76	0.99 (0.73–1.36)	0.97
Sucralose (>0.01 µg/mg)	118 (11.9)	33 (12.1)	1.02 (0.65–1.61)	0.92	0.97 (0.59–1.60)	0.96
Cyclamate (>0.01 µg/mg)	40 (4.1)	10 (3.7)	0.91 (0.44–1.85)	0.79	0.87 (0.40–1.88)	0.72
Steviol glycosides (>0.01 µg/mg)	558 (56.5)	137 (50.4)	0.92 (0.69–1.24)	0.58	1.00 (0.72–1.40)	0.99
Total urinary NNS concentrations (high vs. low)	449 (45.5)	123 (45.2)	1.07 (0.80–1.42)	0.66	1.09 (0.80–1.48)	0.58
**Premenopausal breast cancer (*n* = 38)**						
Aspartame (>0.004 µg/mg)	228 (23.1)	12 (31.6)	1.39 (0.63–3.07)	0.42	1.12 (0.43–2.94)	0.81
Acesulfame (>0.01 µg/mg)	97 (9.8)	5 (13.2)	1.27 (0.45–3.60)	0.65	1.13 (0.30–4.18)	0.86
Saccharin (>0.29 µg/mg)	495 (50.1)	20 (52.6)	1.05 (0.50–2.20)	0.89	1.21 (0.46–3.16)	0.70
Sucralose (>0.01 µg/mg)	118 (11.9)	9 (23.7)	3.39 (1.28–8.96)	0.014	5.90 (1.44–24.20)	0.014
Cyclamate (>0.01 µg/mg)	40 (4.1)	2 (5.3)	1.04 (0.23–4.81)	0.96	1.93 (0.27–13.68)	0.51
Steviol glycosides (>0.01 µg/mg)	558 (56.5)	22 (57.9)	1.19 (0.55–2.56)	0.66	1.81 (0.58–5.64)	0.30
Total urinary NNS concentrations (high vs. low)	449 (45.5)	23 (60.5)	1.71 (0.83–3.54)	0.15	2.44 (0.89–6.72)	0.084
**Postmenopausal breast cancer (*n* = 234)**						
Aspartame (>0.004 µg/mg)	228 (23.1)	60 (25.6)	1.16 (0.81–1.65)	0.42	1.17 (0.80–1.71)	0.42
Acesulfame (>0.01 µg/mg)	97 (9.8)	23 (9.8)	1.16 (0.68–1.98)	0.59	1.08 (0.62–1.90)	0.78
Saccharin (>0.29 µg/mg)	495 (50.1)	125 (53.4)	1.03 (0.76–1.41)	0.83	0.97 (0.69–1.36)	0.86
Sucralose (>0.01 µg/mg)	118 (11.9)	24 (10.3)	0.83 (0.50–1.38)	0.48	0.76 (0.43–1.34)	0.34
Cyclamate (>0.01 µg/mg)	40 (4.1)	8 (3.4)	0.83 (0.38–1.84)	0.65	0.78 (0.33–1.84)	0.57
Steviol glycosides (>0.01 µg/mg)	558 (56.5)	115 (49.2)	0.90 (0.65–1.24)	0.51	0.97 (0.68–1.39)	0.88
Total urinary NNS concentrations (high vs. low)	449 (45.5)	100 (42.7)	0.98 (0.72–1.34)	0.90	0.97 (0.70–1.35)	0.87

Data are expressed as hazard ratios (HRs) with 95% confidence intervals (95% CIs), obtained from multivariable-adjusted weighted Cox regression models accounting for the case-cohort design. Model 1 was adjusted for age and energy intake. Model 2 was additionally adjusted for educational level, housing tenure, marital status, occupational class, place of residence, smoking status, leisure-time physical activity, body mass index, number of chronic diseases, menopausal status, hormone replacement therapy, oral contraceptive use, number of children, family history of breast cancer, and the Mediterranean Diet Score. Menopausal status was not included in the analyses stratified by premenopausal and postmenopausal breast cancer incidence.

**Table 4:** Association of urinary concentrations of sucralose with body mass index and established markers of inflammation, glucose metabolism, and renal function in the subcohort (*n* = 988).

	Sucralose (Presence vs. Absence)
	β (95% CI)
**Body mass index** (kg/m^2^)	−0.13 (−1.09, 0.83)
**Inflammatory markers**	
C-reactive protein, mg/dL	0.09 (−0.44, 0.62)
White blood cell count, ×10^9^/L	0.24 (−0.05, 0.53)
Granulocyte-to-lymphocyte ratio	0.09 (−0.06, 0.23)
**Markers of glucose metabolism in fasting participants ^a^**	
Blood glucose, mg/dL	−5.15 (−8.40, −1.89)
Insulin, pmol/L	4.10 (−1.13, 9.34)
C-peptide, ng/mL	0.11 (−0.01, 0.23)
HOMA-IR	0.04 (−0.19, 0.27)
HOMA-IR > 2.5 (OR; 95% CI)	0.91 (0.47, 1.76)
**Markers of glucose metabolism in fasting participants** **without T2D ^b^**	
Blood glucose, mg/dL	−2.96 (−5.56, −0.36)
Insulin, pmol/L	4.67 (−0.60, 9.95)
C-peptide, ng/mL	0.11 (−0.005, 0.23)
HOMA-IR	0.12 (−0.09, 0.34)
HOMA-IR > 2.5 (OR; 95% CI)	0.97 (0.49, 1.93)
**Markers of renal function**	
Cystatin C, mg/L	0.01 (−0.02, 0.04)
Creatinine, mg/dL	0.01 (−0.01, 0.03)
eGFRcr-cys, mL/min/1.73 m^2^	−1.56 (−3.96, 0.84)
Impaired kidney function ^c^ (OR; 95% CI)	0.52 (0.15, 1.83)

Abbreviations: HOMA-IR, Homeostasis Model Assessment of Insulin Resistance (HOMA-IR); T2D, type 2 diabetes; eGFRcr-cys, estimated glomerular filtration rate calculated by the 2021 Chronic Kidney Disease Epidemiology Collaboration creatinine–cystatin C equation. Data are expressed as regression coefficient beta with 95% confidence intervals (95% CI), obtained from multivariable-adjusted linear regression models including age, energy intake, educational level, housing tenure, marital status, occupational class, place of residence, smoking status, leisure-time physical activity, body mass index (not for analysis testing BMI as dependent variable), number of chronic diseases, menopausal status, hormone replacement therapy, oral contraception use, number of children, family history of breast cancer, and the Mediterranean Diet Score. Odds ratios (ORs) with 95% CI were used to assess the association between urinary NNS concentrations (binary variables) and impaired glucose metabolism or kidney function. ^a^ Available for 983 participants in fasting status at the time of blood drawing. ^b^ Available for 953 participants in fasting status at the time of blood drawing, and free from type 2 diabetes. ^c^ Impaired kidney function was defined as eGFRcr-cys values <60 mL/min/1.73 m^2^.

## Data Availability

The data underlying this article will be shared upon reasonable request to the corresponding author. The data are stored in an institutional repository (https://repository.neuromed.it), and access is restricted by the ethical approvals and the legislation of the European Union.

## Supplementary Materials

The following supporting information can be downloaded at: https://www.mdpi.com/article/10.3390/nu18182958/s1, Supplementary Methods; Figure S1. Flowchart for selection of study participants from the Moli-sani Study, 2005-2010; Moli-sani Study Investigators; Table S1. Analytical validation parameters for the quantitative determination of urinary non-nutritive sweeteners; Table S2. Urinary concentrations, relative contribution to total urinary non-nutritive sweetener concentrations, and main dietary sources of individual sweeteners in the subcohort (*n* = 988) [62]; Table S3. Associations between urinary non-nutritive sweeteners (NNS) and breast cancer risk after Benjamini–Hochberg false discovery rate (FDR) correction, overall and by subtypes; Table S4. Association of urinary concentrations of non-nutritive sweeteners (NNS) with breast cancer risk after additional adjustment for ultra-processed food consumption according to the Nova classification; Table S5. Subgroup analyses for the association between urinary non-nutritive sweeteners (NNS) and breast cancer risk.

## Abbreviations

NNS	Non-nutritive sweeteners
BC	Breast cancer
MDS	Mediterranean Diet Score
FFQ	Food frequency questionnaire
HOMA-IR	Homeostasis Model Assessment of Insulin Resistance
eGFR	Estimated glomerular filtration rate
eGFRcr-cys	Estimated glomerular filtration rate creatinine-cystatin C
MET-h	Metabolic equivalent task-hours
BMI	Body mass index
SD	Standard deviation
HR	Hazard Ratios
95% CI	95% confidence interval
