# Supplementary material for: Urinary Concentrations of Non-Nutritive Sweeteners and Breast Cancer: A Case-Cohort Analysis of the Moli-sani Study
Source: Nutrients. 2026 Sep 9;18(18):2958. doi: 10.3390/nu18182958 (PMC13609978; doi:10.3390/nu18182958)
Supplement: Supplementary file 1 [file nutrients-18-02958-s001.zip › nutrients-4514943-supplementary.pdf]

Urinary concentrations of non-nutritive sweeteners and breast cancer: a case-cohort analysis of the Moli-sani Study

Marialaura Bonaccio, Augusto Di Castelnuovo, Simona Costanzo, Claudia F. Martinez, Emilia Ruggiero, Teresa Panzera, Simona Esposito, Amalia De Curtis, Sara Magnacca, Jacopo Troisi, Chiara Cerletti, Maria Benedetta Donati, Giovanni de Gaetano and Licia Iacoviello

On-line supplementary material.

Supplementary Figure S1. Flowchart for selection of study participants from the Moli-sani Study, 2005-2010.

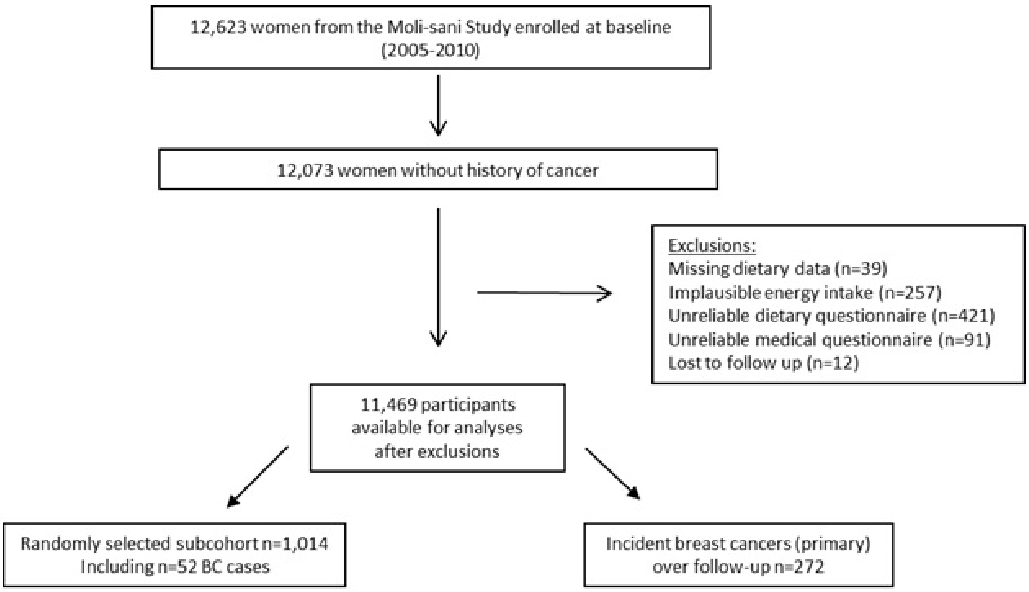

Assessment of urinary excretions of non-nutritive sweeteners in the analytic sample.

Reagents and Chemicals

LC-MS grade solvents, including water, acetonitrile, and formic acid, were obtained from VWR (Radnor, PA, USA). Certified analytical standards of the sweeteners were used. Calibration standards were sourced from TCI (Tokyo Chemical Industry Co., Ltd., Japan) and HWI pharma services (Rülzheim, Germany). Creatinine was measured using a commercial enzymatic assay kit (Abbott Alinity ci-series).

Sample Preparation

For each analysis, 100 µL of urine was mixed with 900 µL of an acetonitrile/water (90:10 v/v) solution containing 0.1% formic acid to precipitate proteins. The mixture was vortexed, centrifuged at 14,000 rpm for 10 minutes at 7°C, and 500 µL of the clear supernatant was transferred into silanized glass vials. A 1 µL aliquot was injected into the LC-MS/MS system for analysis.

Chromatographic Separation

Analyses were performed on a Thermo Scientific Vanquish UHPLC system equipped with a binary pump, autosampler, and column oven. Separation was achieved using an Avantor ACE HILIC column (150 × 2.1 mm, 3 µm particle size). The mobile phase consisted of (A) 0.1% formic acid in water and (B) 0.1% formic acid in acetonitrile. A linear gradient was applied from 98% to 60% B over 4 minutes, followed by a return to initial conditions and re-equilibration, with a total runtime of 7.5 minutes per sample. The flow rate was 0.35 mL/min; column and autosampler temperatures were set at 25°C and 6°C, respectively.

Mass Spectrometry Analysis

Mass spectrometric detection was carried out using a Thermo Q-Exactive Orbitrap hybrid quadrupole mass spectrometer with a heated electrospray ionization (HESI-II) source. The analysis operated in full scan and selected ion monitoring (SIM) modes under both positive and negative ionization conditions. Ion source parameters included sheath gas at 45 units, auxiliary gas at 12 units, capillary temperature at 290°C, and heater temperature at 320°C. Spray voltage was set to +3.3 kV and -2.5 kV for positive and negative modes, respectively. Mass calibration was performed continuously using an internal calibrant.

Calibration, Analytical Validation, and Quality Control

Matrix-matched calibration standards were prepared by fortifying a pooled urine sample previously verified to be free of detectable signals attributable to the analytes of interest. A matrix blank and six non-zero calibration levels (1, 5, 10, 25, 50, and 100 ng/mL) were included. Each non-zero level

was measured in triplicate, yielding n = 18 observations for the linear calibration regression of each quantitatively assessed analyte. Calibration and quality-control solutions were prepared from independent primary and intermediate stock solutions.

Calibration model. For each analyte, the instrumental response (Y; peak area) was modelled as a linear function of concentration (X; ng/mL) according to Y = a + bX, where a is the intercept and b is the slope of the calibration curve. The residual standard deviation of the regression, s(y/x), was calculated from the residuals between observed and fitted responses, and the dispersion of the calibration concentrations was represented by $SSx = \Sigma(X_i - \bar{X})^2$. Linearity was assessed by the coefficient of determination (R^2), which was >0.99 for all quantitatively assessed sweeteners.

$$Y = a + bX$$
$$s(y/x) = [\Sigma(Y_i - \hat{Y}_i)^2 / (n - 2)]^{1/2}$$
$$SSx = \Sigma(X_i - \bar{X})^2$$

Estimation of the analytical limits. LOD and LOQ were derived from the matrix-matched calibration regression and were therefore not defined solely by visual inspection of chromatographic peak detectability. The calculations incorporated the residual variability of the analytical response, the sensitivity of the calibration function through its slope, the number and distribution of calibration observations, and the number of replicate measurements. The calculation implemented in the calibration workbooks used a two-tailed Student's t critical value at $\alpha = 0.05$ with n − 2 degrees of freedom. The number of replicate measurements was $q_r = 3$.

For compactness, the common prediction term K was defined as:

$$K = [1/q_r + 1/n + \bar{X}^2/SSx]^{1/2}$$

A decision limit was first calculated in the response domain. With t denoting the critical Student's t value and s(y/x) the residual standard deviation of the calibration regression, the response-domain decision limit (Ydc) and its corresponding concentration-domain value (Xdc) were calculated as:

$$Ydc = \bar{Y} - b\bar{X} + t \cdot s(y/x) \cdot K$$
$$Xdc = (Ydc - \bar{Y})/b + \bar{X}$$

The response-domain detection limit (Yr) was then obtained by adding a second t-weighted prediction term to the decision limit. Inverse prediction through the calibration equation provided the concentration-domain detection limit (Xr), which was used as the LOD:

$$Yr = Ydc + t \cdot s(y/x) \cdot K$$

$$LOD = Xr = (Yr - \bar{Y})/b + \bar{X}$$

The limit of quantification was operationally obtained from the concentration-domain limit of determination implemented in the calibration workbooks. The response-domain determination limit (Ydt) used a relative term of 0.1 in the denominator, as specified in the calculation template, and was converted to concentration by inverse prediction:

$$Ydt = [s(y/x)/0.1] \cdot K$$
$$LOQ = Xdt = (Ydt - \bar{Y})/b + \bar{X}$$

Thus, both analytical limits were expressed in concentration units after inverse prediction from the calibration model. The analyte-specific values obtained directly from the calibration workbooks are reported in Supplementary Table S1. These calculations are distinct from the subsequent statistical substitution of observations falling below the analytical thresholds.

Quality control, recovery, and repeatability. QC samples were prepared independently from separate primary and intermediate stock solutions at nominal concentrations of 10 and 100 ng/mL. QC samples were randomly interspersed within each analytical batch and accounted for approximately 10% of the samples analysed. Each study sample was analysed in triplicate. Repeatability was additionally monitored using 10 replicate analyses of a fortified urine sample in each batch, and analytical performance was followed using control charts. Recovery was assessed at 10 and 100 ng/mL. Across the quantitatively assessed sweeteners, mean recoveries ranged from 94.3% to 102.7%; analyte-specific values are reported in **Supplementary Table S1**.

Creatinine Normalization

Creatinine levels in urine were quantified using an enzymatic colorimetric assay, allowing normalization of analyte concentrations. The reaction sequence involved enzymatic conversion of creatinine to creatine, followed by formation of a chromophore (quinoneimine), with absorbance measured at 548 nm. Urinary sweetener concentrations were normalized to creatinine (μg/mg creatinine) to reduce variability attributable to urine dilution in spot samples. This normalization was intended to account for differences in urinary concentration and does not correct for compound-specific renal handling.

Statistical Analysis

All analyses were conducted in a blinded fashion. Raw analytical concentrations were reported without correction for analyte recovery, in line with the validation procedure. Results were expressed as both absolute concentrations (μg/L) and creatinine-normalized concentrations (μg/mg creatinine). LOD and LOQ were treated as analytical performance thresholds and not as measured

concentrations. For statistical data handling, measurements below the LOD were replaced by LOD/√2; measurements yielding a detectable signal but falling below the LOQ were analogously replaced by LOQ/√2. These substituted values were imputed solely to permit statistical handling of left-censored observations and were not interpreted as directly quantified analytical concentrations. Normality of the distributions was assessed using the Shapiro-Wilk test.

Supplementary Table S1. Analytical validation parameters for the quantitative determination of urinary non-nutritive sweeteners.

Analyte	Calibration equation (Y = a + bX)	R^2	LOD (ng/mL)	LOQ (ng/mL)	Recovery at 10 ng/mL (%)	Recovery at 100 ng/mL (%)
Acesulfame K	$Y = 5.369\times10^{7} + 1.368\times10^{7}X$	0.99701	5.6	9.3	94.3 ± 5.4	101.3 ± 6.2
Saccharin	$Y = 2.233\times10^{7} + 1.943\times10^{6}X$	0.99637	3.1	6.2	96.2 ± 6.1	94.7 ± 8.4
Aspartame	$Y = -2.013\times10^{6} + 8.831\times10^{5}X$	0.99888	3.4	10.4	98.6 ± 2.1	97.7 ± 4.8
Cyclamate	$Y = 7.913\times10^{7} + 1.616\times10^{7}X$	0.99253	8.9	16.1	95.2 ± 5.7	102.7 ± 10.3
Stevioside*	$Y = -1.397\times10^{5} + 4.148\times10^{5}X$	0.99493	7.3	17.6	95.3 ± 7.9	99.7 ± 11.2
Sucralose	$Y = 2.246\times10^{6} + 1.381\times10^{6}X$	0.99589	6.6	13.9	97.8 ± 7.3	101.4 ± 9.9

A matrix blank and calibration levels of 1, 5, 10, 25, 50, and 100 ng/mL were used; the six non-zero levels were measured in triplicate for regression analysis. LOD and LOQ values shown here were obtained directly from the corresponding calibration workbooks using the regression-based calculations described above. *Stevioside was used for quantitative assessment of steviol glycosides; additional steviol glycosides were assessed qualitatively.

Supplementary Table S2. Urinary concentrations, relative contribution to total urinary non-nutritive sweetener concentrations, and main dietary sources of individual sweeteners in the subcohort (n=988).

Non-nutritive sweeteners (creatinine-adjusted; μg/mg)	Mean	Median	Std Dev	Minimum	Maximum	Lower Quartile	Upper Quartile	Contribution to total urinary NNS concentration (%)	Main dietary sources in the Italian market [1]
Aspartame	0.015476	0.004307	0.092584	0.004307	2.52698	0.004307	0.004307	3.2%	Non-alcoholic beverages; table-top sweeteners; food supplements
Acesulfame K	0.017629	0.007094	0.057508	0.007094	1.187657	0.007094	0.007094	3.6%	Non-alcoholic beverages; table-top sweeteners; food supplements
Saccharin	0.371823	0.294285	0.292278	0.007854	2.012899	0.153099	0.531297	76.7%	Table-top sweeteners; food supplements; jams, jellies and marmalades
Sucralose	0.012406	0.008361	0.015329	0.008361	0.236922	0.008361	0.008361	2.6%	Non-alcoholic beverages; table-top sweeteners; food supplements
Cyclamate	0.038313	0.011274	0.304034	0.011274	6.99244	0.011274	0.011274	7.9%	Non-alcoholic beverages; table-top sweeteners; food supplements
Steviol glycosides	0.029208	0.022295	0.02209	0.009247	0.084486	0.009247	0.050394	6.0%	Desserts, particularly yogurts and ice creams

[1] Main dietary sources are based on data from the Italian food market obtained from Le Donne C, Mistura L, Goscinny S, Janvier S, Cuypers K, D'Addezio L, Sette S, Catasta G, Ferrari M, Piccinelli R, Van Loco J, Turrini A. Assessment of dietary intake of 10 intense sweeteners by the Italian population. Food Chem Toxicol. 2017 Apr;102:186-197. doi: 10.1016/j.fct.2017.02.014.

Supplementary Table S3. Associations between urinary non-nutritive sweeteners (NNS) and breast cancer risk after Benjamini–Hochberg false discovery rate (FDR) correction, overall and by subtypes.

	Overall BC		Premenopausal BC		Postmenopausal BC	
Non-nutritive sweeteners	P-value	FDR-adjusted P-value (q-value)	P-value	FDR-adjusted P-value (q-value)	P-value	FDR-adjusted P-value (q-value)
Aspartame	0.26	0.99	0.81	0.86	0.42	0.88
Acesulfame	0.65	0.99	0.86	0.86	0.78	0.88
Saccharine	0.97	0.99	0.70	0.86	0.86	0.88
Sucralose	0.96	0.99	0.014	0.10	0.34	0.88
Cyclamate	0.72	0.99	0.51	0.86	0.57	0.88
Steviol glycosides	0.99	0.99	0.30	0.70	0.88	0.88
Total urinary NNS concentrations	0.58	0.99	0.084	0.29	0.88	0.88

P-values were obtained from multivariable-adjusted weighted Cox regression models accounting for the case-cohort design. Models were adjusted for age, energy intake, educational level, housing tenure, marital status, occupational class, place of residence, smoking status, leisure-time physical activity, body mass index, number of chronic diseases, menopausal status, hormone replacement therapy, oral contraceptive use, number of children, family history of breast cancer, and Mediterranean Diet Score. Menopausal status was not included in the analyses stratified by premenopausal and postmenopausal breast cancer incidence.

Multiple testing correction was performed using the Benjamini–Hochberg false discovery rate procedure. FDR-adjusted P-values (q-values) <0.05 were considered statistically significant.

Supplementary Table S4. Association of urinary concentrations of non-nutritive sweeteners (NNS) with breast cancer risk after additional adjustment for ultra-processed food consumption according to the Nova classification.

Urinary NNS	Subcohort, n (%)	BC cases, n (%)	HR (95% CI)	P-value
**Overall breast cancer (n=272)**				
Aspartame (>0.004 µg/mg)	228 (23.1)	72 (26.5)	1.21 (0.86-1.72)	0.27
Acesulfame (>0.01 µg/mg)	97 (9.8)	28 (10.3)	1.12 (0.67-1.87)	0.67
Saccharin (>0.29 µg/mg)	495 (50.1)	145 (53.3)	1.00 (0.73-1.37)	0.98
Sucralose (>0.01 µg/mg)	118 (11.9)	33 (12.1)	0.96 (0.58-1.60)	0.88
Cyclamate (>0.01 µg/mg)	40 (4.1)	10 (3.7)	0.87 (0.40-1.87)	0.71
Steviol glycosides (>0.01 µg/mg)	558 (56.5)	137 (50.4)	1.00 (0.72–1.40)	0.98
Total urinary NNS concentrations (high vs. low)	449 (45.5)	123 (45.2)	1.09 (0.80–1.47)	0.60
**Premenopausal breast cancer (n=38)**				
Aspartame (>0.004 µg/mg)	228 (23.1)	12 (31.6)	1.13 (0.43–2.93)	0.81
Acesulfame (>0.01 µg/mg)	97 (9.8)	5 (13.2)	1.14 (0.31–4.18)	0.84
Saccharin (>0.29 µg/mg)	495 (50.1)	20 (52.6)	1.21 (0.46–3.17)	0.70
Sucralose (>0.01 µg/mg)	118 (11.9)	9 (23.7)	5.89 (1.44–24.13)	0.014
Cyclamate (>0.01 µg/mg)	40 (4.1)	2 (5.3)	2.00 (0.28–14.06)	0.49
Steviol glycosides (>0.01 µg/mg)	558 (56.5)	22 (57.9)	1.81 (0.58–5.66)	0.31
Total urinary NNS concentrations (high vs. low)	449 (45.5)	23 (60.5)	2.44 (0.89–6.72)	0.084
**Postmenopausal breast cancer (n=234)**				
Aspartame (>0.004 µg/mg)	228 (23.1)	60 (25.6)	1.17 (0.80–1.71)	0.43
Acesulfame (>0.01 µg/mg)	97 (9.8)	23 (9.8)	1.07 (0.61–1.91)	0.81
Saccharin (>0.29 µg/mg)	495 (50.1)	125 (53.4)	0.97 (0.69–1.36)	0.86
Sucralose (>0.01 µg/mg)	118 (11.9)	24 (10.3)	0.76 (0.43–1.33)	0.33
Cyclamate (>0.01 µg/mg)	40 (4.1)	8 (3.4)	0.78 (0.33–1.83)	0.56
Steviol glycosides (>0.01 µg/mg)	558 (56.5)	115 (49.2)	0.97 (0.68–1.39)	0.88
Total urinary NNS concentrations (high vs. low)	449 (45.5)	100 (42.7)	1.00 (0.98-1.03)	0.75

Data are expressed as hazard ratios (HRs) with 95% confidence intervals (95% CIs), obtained from multivariable-adjusted weighted Cox regression models accounting for the case-cohort design. The model was adjusted for age, energy intake, educational level, housing tenure, marital status, occupational class, place of residence, smoking status, leisure-time physical activity, body mass index, number of chronic diseases, menopausal status, hormone replacement therapy, oral contraceptive use, number of children, family history of breast cancer, Mediterranean Diet Score, and ultra-processed food consumption, defined according to the Nova classification and expressed as percentage of total energy intake. Menopausal status was not included in the analyses stratified by premenopausal and postmenopausal breast cancer incidence.

Supplementary Table S5. Subgroup analyses for the association between urinary non-nutritive sweeteners (NNS) and breast cancer risk.

Urinary non-nutritive sweetener	**Overall HR**	**BMI ≤ 30 kg/m²**	**BMI > 30 kg/m²**	**No family history of BC**	**Aged ≤53 y**	**Aged >53 y**
N of cases/n of participants	272/1234	185/857	87/377	248/1165	117/601	155/633
Aspartame	1.24 (0.86-1.79)	1.32 (0.87-2.14)	1.01 (0.49-2.07)	1.23 (0.85-1.79)	1.25 (0.72-2.18)	1.25 (0.74-2.12)
Acesulfame	1.11 (0.67-1.66)	1.17 (0.64-2.13)	1.03 (0.32-3.36)	1.08 (0.63-1.84)	0.68 (0.31-1.49)	1.88 (0.91-3.90)
Saccharine	1.05 (0.73-1.51)	1.09 (0.69-1.74)	1.07 (0.51-2.25)	1.01 (0.70-1.45)	1.04 (0.60-1.78)	1.05 (0.62-1.79)
Sucralose	0.94 (0.57-1.56)	0.76 (0.40-1.45)	1.65 (0.59-4.60)	1.05 (0.62-1.77)	1.38 (0.64-2.99)	0.75 (0.37-1.53)
Cyclamate	0.83 (0.38-1.82)	1.05 (0.39-2.80)	0.46 (0.08-2.55)	0.88 (0.39-2.02)	0.84 (0.25-2.86)	0.91 (0.28-3.00)
Steviol glycosides	1.01 (0.70-1.46)	1.00 (0.64-1.55)	1.09 (0.50-2.37)	0.95 (0.65-1.39)	0.97 (0.57-1.67)	0.94 (0.54-1.64)
Total NNS	1.09 (0.80-1.48)	1.11 (0.76-1.61)	1.11 (0.61-2.02)	1.09 (0.79-1.49)	1.14 (0.71-1.84)	1.01 (0.66-1.55)

Data are presented as hazard ratios (HRs) and 95% confidence intervals (95% CIs) estimated from multivariable-adjusted Cox proportional hazards models using weighted Cox regression models for the case-cohort design. Models were adjusted for age (except in age-stratified analyses), total energy intake, educational level, housing tenure, marital status, occupational class, place of residence, smoking status, leisure-time physical activity, body mass index (except in BMI-stratified analyses), number of chronic diseases, menopausal status, hormone replacement therapy, oral contraceptive use, number of children, family history of breast cancer (except in analyses stratified by family history), and the Mediterranean Diet Score. Individual NNS were mutually adjusted for by including all urinary NNS simultaneously in the same model.

Moli-sani Study Investigators

The enrolment phase of the Moli-sani Study was conducted at the Research Laboratories of the Catholic University in Campobasso (Italy), the follow up of the Moli-sani cohort is being conducted at the Research Unit of Epidemiology and Prevention of the IRCCS Neuromed, Pozzilli, Italy.

Steering Committee: Licia Iacoviello*# (Chairperson), Giovanni de Gaetano*, Maria Benedetta Donati*.

Scientific Secretariat: Chiara Cerletti* (Coordinator), Marialaura Bonaccio*, Americo Bonanni*, Simona Costanzo*°, Amalia De Curtis*, Augusto Di Castelnuovo*, Alessandro Gialluisi*#, Francesco Gianfagna°, Mariarosaria Persichillo*.

Safety and Ethical Committee: Jos Vermylen (Catholic University, Leuven, Belgio) (Chairperson), Renzo Pegoraro (Pontificia Accademia per la Vita, Roma, Italy), Antonio G. Spagnolo (Catholic University, Roma, Italy).

External Event Adjudicating Committee: Deodato Assanelli (Brescia, Italy), Livia Rago (Campobasso, Italy).

Baseline and Follow-up Data Management: Simona Costanzo*° (Coordinator), Sabatino Orlandi*, Teresa Panzera*.

Data Analysis: Augusto Di Castelnuovo* (Coordinator), Marialaura Bonaccio*, Francesca Bracone*, Simona Costanzo*°, Giuseppe Di Costanzo*, Simona Esposito*, Alessandro Gialluisi*#, Anwal Ghulam°, Francesco Gianfagna°, Martina Morelli*†, Maria Loreto Muñoz Venegas*†, Antonietta Pepe*, Emilia Ruggiero*§.

Biobank, Molecular and Genetic Laboratory: Amalia De Curtis* (Coordinator), Concetta Civitillo*†, Alisia Cretella*†, Sara Magnacca*.

Recruitment Staff: Mariarosaria Persichillo* (Coordinator), Francesca Bracone*, Giuseppe Di Costanzo*, Martina Morelli*†.

Communication and Press Office: Americo Bonanni*.

Regional Institutions: Direzione Generale per la Salute - Regione Molise; Azienda Sanitaria Regionale del Molise (ASReM, Italy); Agenzia Regionale per la Protezione Ambientale del Molise (ARPA Molise, Italy); Molise Dati Spa (Campobasso, Italy); Offices of vital statistics of the Molise region.

Hospitals: Presidi Ospedalieri ASReM: Ospedale A. Cardarelli – Campobasso, Ospedale F. Veneziale – Isernia, Ospedale San Timoteo - Termoli (CB), Ospedale Ss. Rosario - Venafro (IS), Ospedale Vietri – Larino (CB), Ospedale San Francesco Caracciolo - Agnone (IS); Casa di Cura Villa Maria - Campobasso; Responsible Research Hospital - Campobasso; IRCCS Neuromed - Pozzilli (IS).

**Research Unit of Epidemiology and Prevention, IRCCS Neuromed, Pozzilli, Italy*
#Department of Medicine and Surgery, LUM University "Giuseppe Degennaro", Casamassima, Italy
°Department of Medicine and Surgery, University of Insubria, Varese, Italy
§Fellow of the Fondazione Umberto Veronesi, Italy
†Fondazione Veronesi – Piattaforma UMBERTO

Moli-sani Study Past Investigators are available at https://www.moli-sani.org/?page_id=173
